# The *ea22* gene of lambdoid phages: preserved prolysogenic function despite of high sequence diversity

**DOI:** 10.1007/s11262-020-01734-8

**Published:** 2020-01-22

**Authors:** Aleksandra Dydecka, Sylwia Bloch, Agnieszka Necel, Gracja Topka, Alicja Węgrzyn, Jinge Tong, Logan W. Donaldson, Grzegorz Węgrzyn, Bożena Nejman-Faleńczyk

**Affiliations:** 1 Department of Molecular Biology, Faculty of Biology, University of Gdańsk, Wita Stwosza 59, 80-308 Gdańsk, Poland; 2 Department of Biology, York University, 4700 Keele Street, Toronto, ON M3J 1P3 Canada; 3 Laboratory of Molecular Biology, Institute of Biochemistry and Biophysics, Polish Academy of Sciences, Kładki 24, 80-822 Gdańsk, Poland

**Keywords:** *Exo-xis* region, Lambdoid phages development, Shiga toxin-producing *Escherichia coli* (STEC)

## Abstract

The *exo-xis* region of lambdoid phages contains open reading frames and genes that appear to be evolutionarily important. However, this region has received little attention up to now. In this study, we provided evidence that *ea22*, the largest gene of this region, favors the lysogenic pathway over the lytic pathway in contrast to other characterized *exo-xis* region genes including *ea8.5*, *orf61, orf60a*, and *orf63.* Our assays also suggest some functional analogies between Ea22 and the phage integrase protein (Int). While it is unsurprising that Ea22 operates similarly in both λ and Stx phages, we have observed some distinctions that may arise from considerable sequence dissimilarity at the carboxy termini of each protein.

## Introduction

The recognition of Shiga toxin-producing *Escherichia coli* (STEC) bacteria as a public health problem took place in 1982 during an outbreak of hemorrhagic colitis (HC) tied to the consumption of contaminated hamburgers [1]. In 2011, the Shiga toxin-producing *E. coli* serotype O104:H4 was responsible for a serious epidemic in twelve European countries [2, 3].

The main virulence factors of STEC are Shiga toxins (Stx toxins), encoded by genes *stx1* and *stx2* located in genomes of bacteriophages that infect these bacteria [4]. Shiga toxin-converting bacteriophages (Stx phages) belong to the lambdoid family of phages, of which bacteriophage λ is the best investigated member [4–9]. All lambdoid phages indicate high similarities in the life cycle and organization of the genome. Depending on the intracellular conditions, lambdoid phages follow either a lysogenic or lytic developmental pathway leading to dormancy or the production of new viral particles, respectively [10–12]. The effective production of Shiga toxins and their release in human intestine occurs only upon prophage induction and its further lytic development [13–15] suggesting that a therapeutic route may be found by studying the lysogenic-lytic decision further.

Despite over sixty years of research, not all genes in bacteriophage λ have been explored [16]. In other model systems, sequence comparisons are usually enough to fill in any knowledge gaps; however, in bacteriophage λ, this approach suffers due to a lack of similarity to reference proteins [17, 18]. Shiga toxin-converting bacteriophage Ф24_B_ that has rather small genome size (approximately 60 kb) carry 55% of sequences predicted to encode hypothetical proteins. Surprisingly, in its host, *Escherichia coli* bacterium (with genome of about 4600 kb), such sequences represent only 24% and the difference is even more remarkable when sizes of the both genomes are taken into consideration [19]. An imbalance between the amount of functional genomics studies conducted on Stx phages and their host bacteria, makes the functional characterization of Stx phage genomes challenging.

**Fig. 1:**
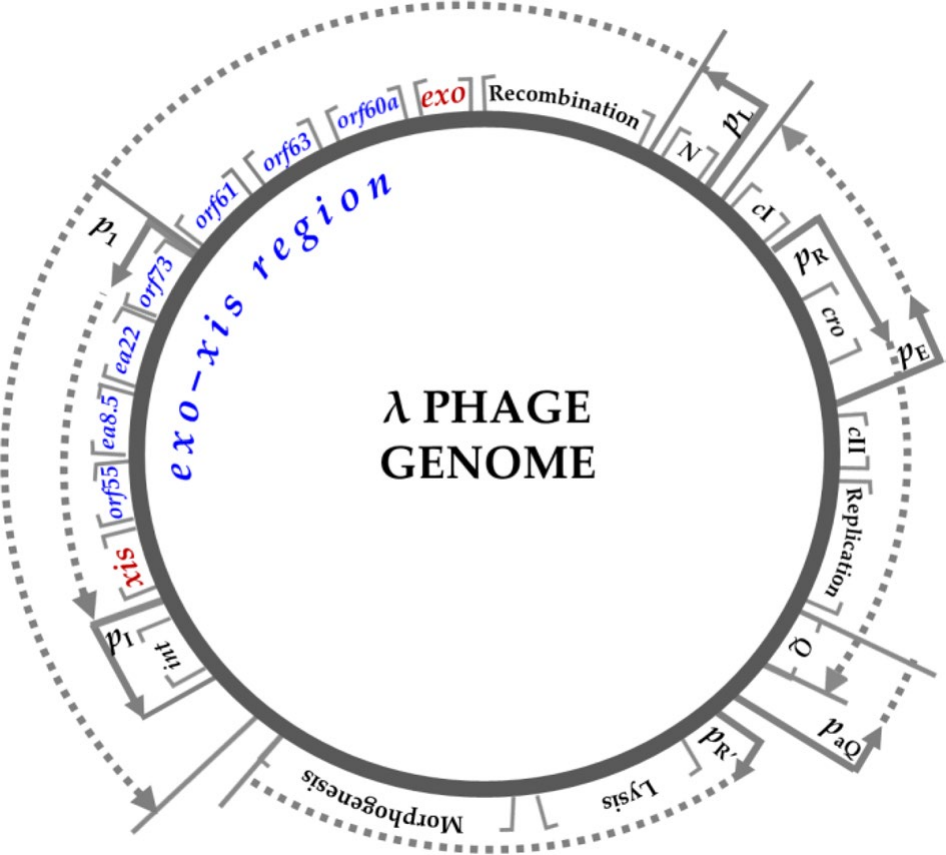
Circular genome map of λ phage highlighting phage genes and promoters of the *exo-xis* region. Promoters and terminators, including newly identified sequence *p*1 that is directly relevant to this study, are depicted by arrows and vertical lines, respectively

Among all lambdoid bacteriophages, the *exo-xis* gene region remained unknown for long time, not only in terms of the hypothetical proteins encoded within it but also their functional attributes. The early *p*L promoter, one of two major promoters repressed by cI protein during lysogeny, is also responsible for expression of *exo-xis* region genes [8, 16, 18]. In λ phage, the *exo-xis* region consists of three already tested open reading frames (ORFs) designated as *orf60a, orf63*, and *orf61* and two recognized genes called *ea22* and *ea8.5* (Fig. 1). Except *ea8.5,* all of them as well as some other uncharacterized ORFs are found in Stx phages [20].

Genes within the *exo-xis* region were previously observed to repress transcription from λ cII-dependent promoters: *p*E, *p*I and *p*aQ that are known to promote lysogenic pathway [21]. Using a multicopy plasmid approach, Bloch and collaborators later revealed new features of *exo-xis* region including impaired lysogenization and more effective induction of prophages, both spontaneous and stimulated by various agents [22]. Furthermore, Licznerska and co-workers demonstrated that in the absence of the *exo-xis* region, the expression of critical Stx phage genes directing lytic development was decreased after prophage induction [23]. Together, these observations demonstrate that lambdoid phage development may be more complex than it was thought previously and that the *exo-xis* region serves a role in this process.

The analysis of individual genes of the *exo-xis* region has been instructive. For example, the *ea8.5* gene contributed to significant repression of *p*aQ, a minor effect on *p*I, and negligible effect on the *p*E promoter [21]. These results suggested that *ea8.5* affects the *p*I and *p*aQ promoters, though its influence is relevantly weaker than the whole *exo-xis* region. It seems that the *ea8.5* product may be partially responsible for the negative regulation of *p*I and *p*aQ. However, the activity of these promoters also depends on at least one other factor located between *exo* and *xis* [21]. The structure of Ea8.5 is a hybrid of two common folds, a homeodomain and a zinc finger, each with a potential nucleic acid binding and protein–protein interaction function [24]. Apart from Ea8.5, the only other *exo-xis* region gene product that has been analyzed biochemically is Orf63, a small oligomeric protein consisting of two alpha helices. Functionally, *orf63* represses expression of the major phage genes responsible for prophage induction thereby delaying the time and decreasing the efficiency of this process [25]. While gene products of *orf60a* and *orf61* remain essentially uncharacterized from a biochemical viewpoint, they do serve important roles in the control of phage lytic development since their deletions significantly delay the induction of Stx prophage treated with hydrogen peroxide and reduce the efficiency of such process. Furthermore, deletion of either *orf60a* or *orf61* enhances post-infection bacterial survival and promotes the formation of lysogens. As was observed for orf63, the effects of *orf60a* or *orf61* deletions were more pronounced in the Stx phage Φ24_B_ than in λ [26].

Our preliminary research on *ea22* showed that deletion of this gene in both λ and Φ24_B_ genomes did not affect phage titer, but promoted a rapid induction of mutant prophages after stimulation by UV irradiation. For other inducing agents, however, the effects were slight or absent [23]. Thus, we hypothesize that *ea22* may play a different function during phage life cycle than other already studied *exo-xis* region genes.

## Materials and methods

### Bioinformatics

The translated amino acid sequences of ea22 from *λ* (NC_001416) and from three Stx phages: Φ24_B_ (HM208303), 933W (NC_000924), and P27 (KU238067) were submitted to the QUICK2D utility for secondary structural prediction, the CLUSTAL utility for alignment, and the MARCOIL/PCOILS utilities for coiled-coil prediction, all hosted on the HHPred server at https://toolkit.tuebingen.mpg.de [27]. The hairpin RNA structures were predicted using Mfold web server available at https://unafold.rna.albany.edu/?q=mfold*.* The RNA–RNA interaction was identified using IntaRNA software available at https://rna.informatik.uni-freiburg.de/IntaRNA.

### Bacterial strains and bacteriophages

The bacterial strains and bacteriophages used in this work are presented in Table 1. Strains with deletion of gene *ea22* or *vb_24B_6c* were constructed by using the λRed homologous recombination system following the procedure described previously [23]. Each deletion was confirmed by DNA sequencing (Genomed) and showed that there was no sequence changes in the other open reading frames from that region. Lysates of lambdoid bacteriophages were stored in TM buffer (10 mM Tris–HCl, 10 mM MgSO_4_, pH 7.2) at 4 °C.

**Table 1:** *Escherichia coli* strains and bacteriophages

Bacterial strains and bacteriophages	Genotype or relevant characteristics	References
*E. coli *strains		
MG1655	F^–^ λ^–^*ilvG rfb-50 rph-1*	[43]
MG1655 (λ)	MG1655 bearing λ prophage	[22]
MG1655 (λΔ*ea22*)	MG1655 bearing λΔ*ea22* prophage	[23]
MG1655 (Φ24_B_)	MG1655 bearing Φ24_B_Δs*tx2*::*cat* prophage	[22]
MG1655 (Φ24_B_Δ*ea22*)	MG1655 bearing Φ24_B_Δs*tx2*::*cat* Δ*vb_24B_6c* prophage	[23]
MG1655 (933W)	MG1655 bearing 933WΔs*tx2*::*catGFP* prophage	This work
MG1655 (P22)	MG1655 bearing P22Δs*tx2*::*catGFP* prophage	This work
MG1655 (P27)	MG1655 bearing P27Δs*tx2*::*catGFP* prophage	This work
MG1655 (P32)	MG1655 bearing P32Δs*tx2*::*catGFP* prophage	This work
Bacteriophages		
λ	Carries a frameshift mutation relative to Ur-lambda	[44]
λΔ*ea22*	λ phage with deletion of *ea22* gene	[23]
Φ24_B_	Φ24_B_Δs*tx2*::*cat* in which *stx* genes were replaced with a chloramphenicol resistance gene (*cat*)	[45]
Φ24_B_Δ*ea22*	Φ24_B_ phage with deletion of *vb_24B_6c*, the homolog of λ *ea22* gene	[23]
933W	933WΔs*tx2*::*catGFP* in which *stx* genes were replaced with a chloramphenicol resistance gene (*cat*) and gene of green fluorescent protein (*GFP*)	[46]
P22	22Δs*tx2*::*catGFP* in which *stx* genes were replaced with a chloramphenicol resistance gene (*cat*) and gene of green fluorescent protein (*GFP*)	[46]
P27	27Δs*tx2*::*catGFP* in which *stx* genes were replaced with a chloramphenicol resistance gene (*cat*) and gene of green fluorescent protein (*GFP*)	[46]
P32	32Δs*tx2*::*catGFP* in which *stx* genes were replaced with a chloramphenicol resistance gene (*cat*) and gene of green fluorescent protein (*GFP*)	[46]

### Growth conditions and bacteriological media

All experiments were performed in the Luria–Bertani (LB; Lab Empire) medium at 30 °C with agitation. The same LB medium, supplemented with 1.5% bacteriological agar (BTL), was used as a bottom agar. The adsorption of phage particles onto the surface of the host cells was conducted by the addition of 10 mM MgSO_4_ (phage λ; Chempur) or 10 mM MgSO_4_ and 10 mM CaCl_2_ (Stx phages; Chempur) to the liquid LB medium. Titration of phage λ and its derivative was carried out by a standard double-layer agar assay with the top agar consisting of LB medium with 0.7% (w/v) bacteriological agar (BTL). The visualization of the plaques of Stx phages was analogous to the standard phage titration procedure, but additionally the bottom agar was supplemented with 2.5 µg/mL of chloramphenicol (Sigma-Aldrich) [28]. Plates were incubated at 37 °C overnight.

### Transcriptional analysis of tested genes by quantitative real-time reverse transcription PCR (qRT-PCR) after bacterial infection with lambdoid phages

Bacterial host *E. coli* MG1655 was grown with shaking to OD of 0.3 (A600 nm) at 30 °C and from it, a 12 mL of sample was collected and centrifuged at 2000×*g* for 10 min at 4 °C. The pellet was washed once with 0.85% NaCl (Chempur) and suspended in 3 mL of LB supplemented with 10 mM MgSO_4_ and 10 mM CaCl_2_. After cooling the mixture on ice, phage particles were added to the bacterial sample at an m.o.i. of 1 and incubated for 30 min at 4 °C. Following this treatment, infected bacteria were transferred to a shaking incubator at 30 °C. After an appropriate period, 10^9^ bacterial cells were harvested and treated with 10 mM NaN_3_ to inhibit the growth of the host. Total RNA was isolated using the High Pure RNA Isolation Kit (Roche Applied Science) following treatment with 2 U of TURBO DNase (Life Technologies) for 60 min at 37 °C as recommended by the manufacturer. The quantity of the total RNA sample was determined by UV absorbance using a Nanodrop spectrophotometer (Eppendorf) and the quality was determined electrophoretically. A cDNA preparation was obtained following the vendor’s protocol using 1.25 µg of RNA template, Transcriptor reverse transcriptase and random hexamer primers (Roche Applied Science). The cDNA preparation was then diluted tenfold and analyzed by qRT-PCR using a LightCycler 480 Real-Time PCR System [29]. Amplification was performed using following program: 95 °C for 5 min; 55 cycles of 95 °C for 10 s; 60 °C for 15 s; and 72 °C for 15 s. All oligonucleotide primers, listed in Table 2, were created by Primer3web version 4.0.0. and synthesized by Sigma-Aldrich or Genomed. The isocitrate dehydrogenase *icdA* was selected as a housekeeping gene [30]. The relative changes in gene expression were analyzed with LinRegPCR using the E-Method with efficiency correction [29].

**Table 2:** Primers used for qRT- PCR of *exo-xis* region and associated genes

Primer name	Sequence (5′ → 3′)
pF_λ_Stx_exo pR_λ_Stx_exo	TGCCGTCACTGCATAAACC TCTATCGCGACGAAAGTATGC
pF_λ_ea22 pR_ λ_ea22	GCAGTTCCAGCACAATCGAT AATGCATGACGACTGGGGAT
pF_Φ24_B__ea22 pR_Φ24_B__ea22	TCAGCAACATGGCATTCACT GGTTGGGAAGCTGAGAGTTG
pF_P27_ea22 pR_P27_ea22	ACATCGTAGGGCATACATCTGTT CGGGTTCTCCTTTCCATTTT
pF_933W_P22_P32_ea22 pR_933W_P22_P32_ea22	TCAGCAACATGGCATTCACT AAGCTGCGTGTTGAGCTTG
pF_λ_Stx_orf61 pR_ λ_Stx_orf61	TTAGCCTGACGGGCAATG CCGACATGGGACTTGTTCA
pF_λ_orf63 pR_ λ_orf63	ACCTGGTTTCTCTCATCTGCT GTTAGCCGCATCCCTTTCAC
pF_Stx_Stx_orf63 pR_ Stx_Stx_orf63	GGGTCTCTCTCGTTTGCTTC TAGCCACATCCCTTTCACAA
pF_λ_orf60a pR_ λ_orf60a	GCATACAGCCCCTCGTTTAT CCGAAATCCACTGAAAGCAC
pF_Stx_orf60a pR_ Stx_orf60a	CATACAGCCCCTCGTTTAT CCGAAATCCACTGAAAGCAC
pF_λ_N pR_ λ_N	CTCGTGATTTCGGTTTGCGA AAGCAGCAAATCCCCTGTTG
pF_Stx_N pR_ Stx_N	AGGCGTTTCGTGAGTACCTT TTACACCGCCCTACTCTAAGC
pF_λ_cI pR_ λ_cI	ACCTCAAGCCAGAATGCAGA CCAAAGGTGATGCGGAGAGA
pF_Stx_cI pR_ Stx_cI	TGCTGTCTCCTTTCACACGA GCGATGGGTGGCTCAAAATT
pF_λ_cro pR_ λ_cro	ATGCGGAAGAGGTAAAGCCC TGGAATGTGTAAGAGCGGGG
pF_Stx_cro pR_ Stx_cro	CGAAGGCTTGTGGAGTTAGC GTCTTAGGGAGGAAGCCGTT
pF_λ_Q pR_ λ_Q	TTCTGCGGTAAGCACGAAC TGCATCAGATAGTTGATAGCCTTT
pF_Stx_Q pR_ Stx_Q	GGGAGTGAGGCTTGAGATGG TACAGAGGTTCTCCCTCCCG
pF_λ_R pR_ λ_R	ATCGACCGTTGCAGCAATA GCTCGAACTGACCATAACCAG
pF_Stx_R pR_ Stx_R	GGGTGGATGGTAAGCCTGT TAACCCGGTCGCATTTTTC
pF_*E.coli*_icdA pR_*E.coli*_icdA	CGAAGCGGCTGACTTAATT GTTACGGTTTTCGCGTTGAT

### One-step growth curve

One-step growth experiment was carried out as described previously with only a few modifications [29]. Briefly, bacteria were cultured in LB until early log-phase (2 × 10^8^ CFU/mL). Following that, a 10 mL sample was centrifuged (2000×*g*, 10 min, temp. 4 °C) and the pellet was suspended in 1 mL of LB supplemented with 3 mM NaN_3_ (Sigma-Aldrich). Phage particles were added to *E. coli* MG1655 host to a multiplicity of infection (m.o.i.) of 0.05 and allowed to be adsorbed for 10 min at 30 °C. After incubation, the mixture was diluted tenfold in LB with 3 mM NaN_3_ and centrifuged (2000×*g*, 10 min, 4 °C) to remove unadsorbed phages. This procedure was repeated three times. A 25 µL aliquot of this suspension was added to 25 mL of LB and incubated with shaking at 30 °C. At a series of time points, samples were withdrawn and titrated under permissive conditions. A free bacteriophage count was determined by using a double-layer agar plate method [28]. The burst size of lambdoid bacteriophages (PFU/cell) was calculated as a ratio of phage titer (PFU/mL) and the number of infection centers.

### Survival of* E. coli* bacteria after infection with lambdoid bacteriophages

The number of host bacteria survived after phage infection was estimated according to the procedure described previously with a few minor modifications [31]. *E. coli* MG1655 was cultured to A_600_ = 0.2 in LB at 30 °C. For each experimental repeat, a 1 mL sample was centrifuged (2000×*g*, 10 min, temp. 4 °C) and the pellet was washed twice with 1 mL of freshly prepared TCM buffer (10 mM Tris–HCl, 10 mM MgSO_4_, 10 mM CaCl_2_, pH 7.2). The bacterial pellet was then suspended in 1 mL of TCM buffer and phage lysate was added to a m.o.i. of 1, 5, and 10. After an incubation at 30 °C, serial dilutions of each mixture were prepared in TCM buffer and 40 μL was spread onto plates containing LB supplemented with 1.5% agar. Plates were incubated at 37 °C overnight. The number of viable cells was calculated on the basis of counted colonies. The percentage of surviving bacteria after infection with phage mutants were calculated in relation to control experiment in which bacteria were infected with wild-type λ or Ф24_B_ phages (assumed as 100%).

### Measurement of the number of bacterial lysogens after bacteriophage infection

This assay and an estimate of the efficiency of prophage formation have previously been described [25]. In brief, 96 colonies of bacteria that survived phage infection were cultivated in a multi-well plate containing 200 μL of LB. Bacterial growth proceeded at 37 °C with shaking until an A_600_ of 0.2 was observed. Cultures were then treated with UV light at 50 J/m^2^ for 20 s to induce prophages and incubated with shaking at 37 °C for 2 h. After the UV induction process, putative bacterial lysogens were mixed with 1% (v/v) chloroform and centrifuged at 2000×*g* for 10 min. A plate containing double-layer LB agar was spotted with 2.5 µL of each supernatant. Lysogens were indicated by the appearance of turbid lysis spots after overnight incubation at 37 °C [28]. In the first step, the efficiency of lysogenization was determined as a percentage of lysogens among all tested 96 bacterial colonies (Table 4). Each experiment was repeated three times. Next, the obtained results were used to calculate a percentage of lysogens per infected cell (Fig. 5). The number of cells in the bacterial population infected with one or more phages was determined by the Poisson distribution.

## Results

### Sequence characteristics

**Fig. 2:**
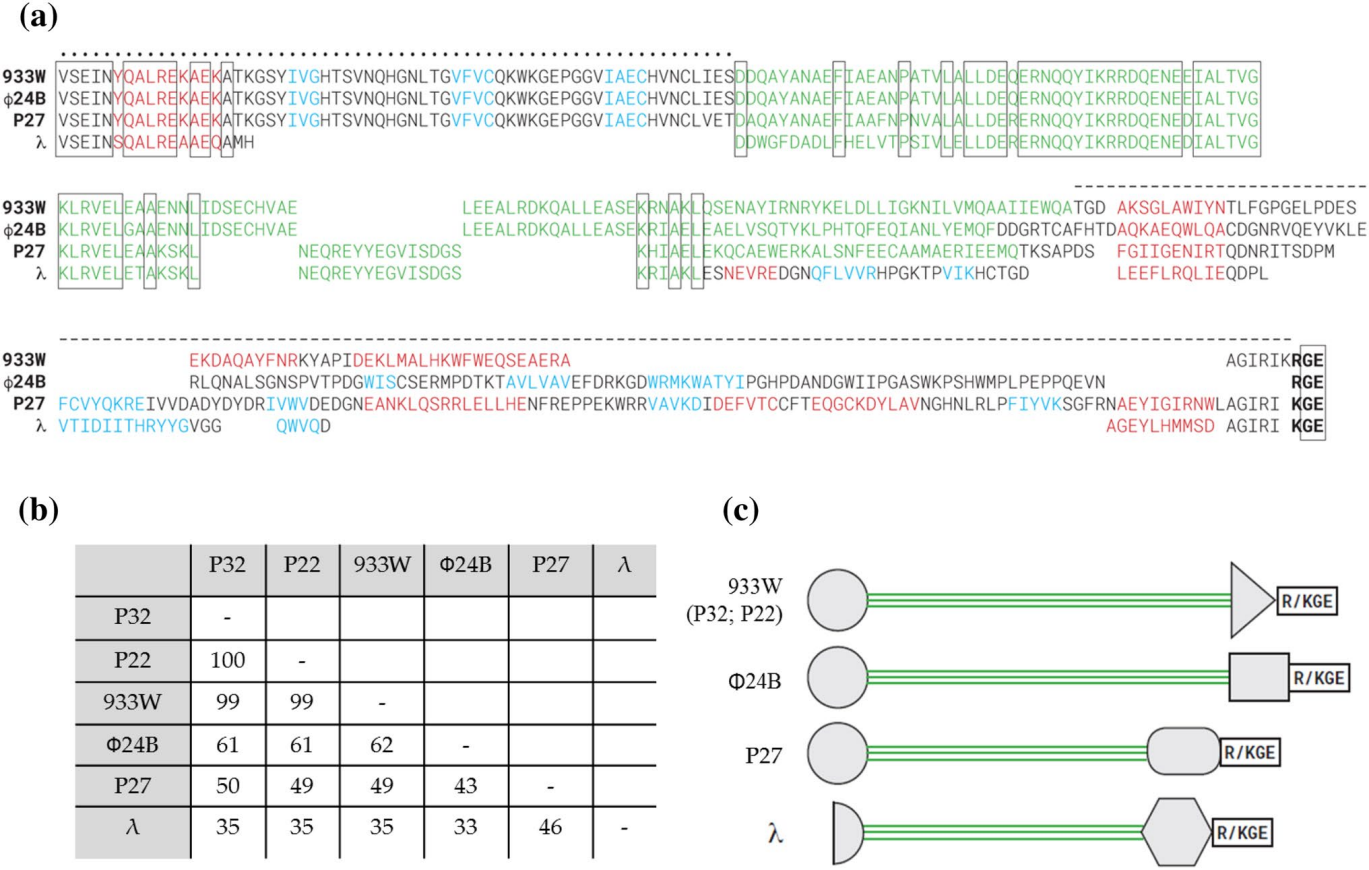
Ea22 sequences and predicted structural characteristics. **a** A multiple sequence alignment of Ea22 from λ (NC_001416) and three Stx phages: Φ24_B_ (HM208303), 933W (NC_000924), and P27 (KU238067). Red, blue, and green coloring indicates predicted helices, strands, and coiled-coil regions, respectively. Since the Ea22 sequences from 933W, P32 (KU238068), and P22 (KU238069) are almost identical, only the sequence from 933W is presented for clarity. Dots and dashes above the alignment denote general boundaries of amino- and carboxy-terminal regions that may be functionally important individually or constitute independent protein domains. **b** Pairwise scores shown as the number of identities between the two sequences, divided by the length of the alignment, and represented as a percentage. **c** A schematic representation of the sequences to highlight the distinctiveness of the amino- and carboxy-terminal regions and a penultimate R/KGE sequence of unknown significance

Since *ea22* is the largest gene in the *exo-xis* region, its gene product presents the opportunity for a comprehensive sequence analysis as shown in Fig. 2a. Among the lambdoid phages selected for this study, Ea22 from the Stx phages P32 and P22 Stx are nearly identical to Ea22 from the Stx phage 933W and demonstrate 99% of similarity (Fig. 2). From a multiple sequence alignment shown in Fig. 2a, the amino-terminal portion is highly conserved among Stx phages but appears to be truncated in λ, leaving only one predicted ά-helix, while the remaining Stx Ea22 proteins have three additional predicted β-strands. Given that the amino-terminal portion represents an autonomously folded domain, as shown schematically by a circle in Fig. 2c, λ *ea22* could possibly be lacking a critical function that is retained in Stx phages. A coiled-coil region follows supported by the prediction of one or more long ά-helices with a characteristic repeating heptad motif of hydrophobic and hydrophilic amino acids at key positions. Among the phages selected for comparison, there is considerable similarity in the first third of the coiled-coil region followed by some variabilities in the middle (one sequence for 933W/Φ24_B_ or another for P27/λ) and more divergent final third that is truncated in the λ Ea22 sequence. While all four Ea22 sequences are predicted to have an internal coiled-coil sequence, it remains unknown what oligomeric state the coiled-coil confers to each expressed protein. The C-terminal portion of each Ea22 sequence appears to be unique to each protein not only in terms of length but also in terms of predicted secondary structures suggesting that each Ea22 protein in this set presents a different domain with possibly different functional consequences. Despite the considerable functional divergence in the C-terminal portion of the sequence, all Ea22 proteins end with a common tripeptide consisting of a basic lysine or arginine followed by a glycine and glutamic acid (+GE) (Fig. 2). The significance of the +GE motif is unknown.

### The *ea22* gene expression during phage infection

**Fig. 3:**
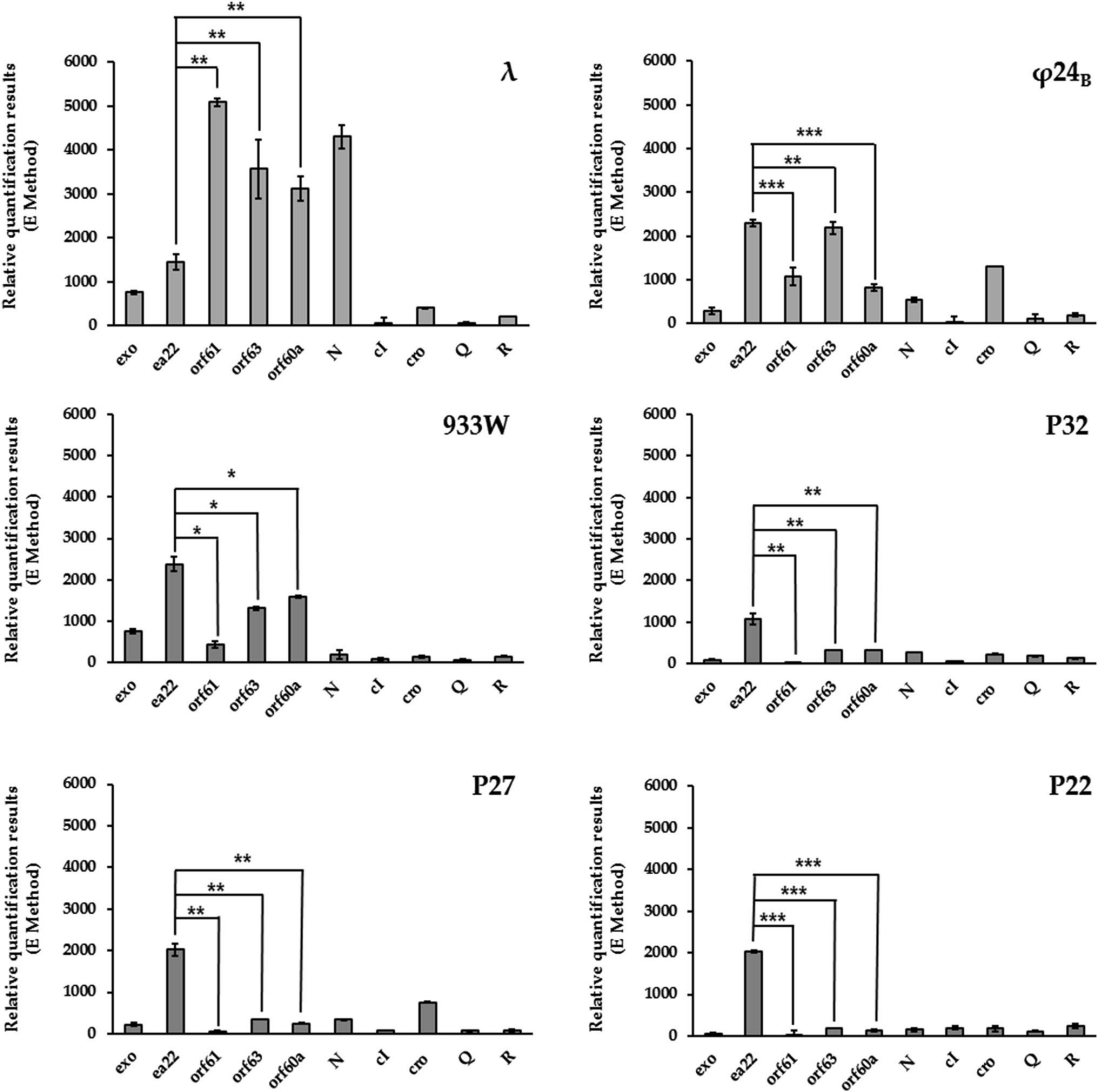
Expression patterns of selected genes measured by RT-qPCR from λ and five Stx phages (Φ24_B_, 933W, P32, P27, P22) upon infection of *E. coli* MG1655 host at m.o.i. of 1. Data represent mean values from three independent experiments with error bars indicating SD. The significance of differences between levels of mRNAs derived from *ea22* and other genes of the *exo-xis* region are observed and marked by asterisks P < 0.05 (*) or P < 0.01 (**) or P < 0.001 (***)

Our analysis of *ea22* began with a comparison of the expression levels between it and a set of genes that includes previously analyzed *exo-xis* region genes and other phage genes that are active during infection (Fig. 3). In accordance with our earlier studies [26, 29], all bacterial cultures were grown at 30 °C to optimize phage adsorption and DNA injection. Levels of mRNAs were measured by RT-qPCR 15 min after λ phage infection and 35 min after infection with Stx phages (Φ24_B_, 933W, P32, P27, P22). These post-infection times were chosen according to our prior knowledge of development of these phages in *E. coli* bacteria [26, 32]. As shown in Fig. 3, differences in the levels of phage mRNAs were observed between *ea22* and *exo-xis* genes *orf61, orf63*, or *orf60a*. In the case of λ phage, the expression of *ea22* is significantly lower than other tested genes from the *exo-xis* region. The opposite outcome, however, was observed for the Stx phages, and notably, *ea22* expression level was the highest among all phage genes tested. The lower expression of both early (*N*, *cI, cro*) as well as middle or late phage genes (*Q, R*), than this observed in *ea22,* may suggest that *ea22* is active during the earliest stages of phage infection. This effect is particularly evident in phages P32, P27, and P22 in which the level of *ea22* expression significantly exceeds the low and surprisingly equal level of expression of other analyzed genes. Our previous report indicated that the tested phage genes, both early and late, revealed different levels of expression which increased during the time-course infection experiment [29]. In that infection assay, which differ in the applied m.o.i. = 5 [29], the expression of early genes *N* and *cro* reached the highest values in 10–20 min after infection with λ, or in 30–40 min after infection with Stx phage Φ24_B_. Importantly, Φ24_B_ showed kinetics of development, following infection of *E. coli* cells, similar to those of 933W, P32, P27, and P22 [32]. In this light, the equal level of expression of the studied genes (except *ea22*), observed in this work, may indicate that their efficient expression have not started yet under these conditions.

### Effect of *ea22* deletion on phage development and host survival

To be consistent with previous developmental studies, that we have performed on *exo-xis* region genes [25, 26], we also selected λ and Φ24_B_ as representative Stx^–^ (λ) or Stx^+^ lambdoid phages in this report. Importantly, these two phages show the highest Ea22 sequence divergency (Fig. 2) and among the Stx^+^ phages, Φ24_B_ offers the practical benefits of stable titer of the phage lysate during storage, in addition to developing efficiently in *E. coli* bacteria at 30 °C. A growth temperature 30 °C was followed according to our previous studies [25, 26, 29] to slow down bacterial metabolism and the rate of phage propagation.

In a ‘one-step’ growth experiment, we observed that both λ and Φ24_B_ phages lacking the *ea22* gene presents shorter latent period, however, produced more progeny per infected cell and thus caused a more rapid lysis of the bacterium than their wild-type counterparts. These distinctions were more pronounced for phage Φ24_B_, suggesting that Stx phages may possess additional factors that synergize the effects of *ea22* (Table 3).

**Table 3:** Parameters of the intracellular development of wild-type phages or their *ea22* deletion mutants in *E. coli* MG1655 bacteria

Strain/phage	Latent period	Burst size^a^
MG1655+λ	35 min	68 ± 15
MG1655+λ∆*ea22*	30 min	96 ± 23
MG1655+Ф24_B_	135 min	25 ± 7
MG1655+Ф24_B_∆*ea22*	85 min	110 ± 33

^a^The burst size of lambdoid bacteriophages (PFU/cell) was calculated as a ratio of phage titer (PFU/mL) and the number of infection centers. The presented values were estimated at 90 min for λ or at 180 min for Ф24_B_ of phages development in *E. coli* bacteria

**Fig. 4:**
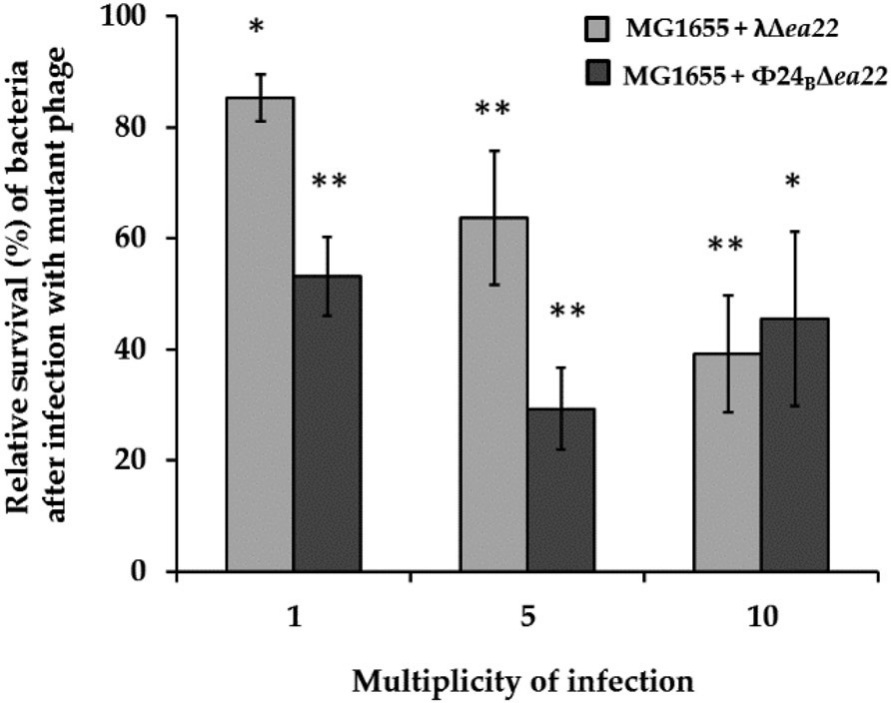
Survival (%) of *E. coli* strain MG1655 after infection with deletion mutants λΔ*ea22* (light gray columns) or Φ24Δ*ea22* (dark gray columns) in relation to bacteria that survived the infection with wild-type λ or Φ24 phages (control experiment assumed as 100%). Experiments were performed at three m.o.i. levels. Results are shown as mean values ± SD from three independent experiments. The significance of the differences between fractions of bacterial cells infected with wild-type phages and their *ea22* deletion mutants are marked by asterisks, P < 0.05 (*) or P < 0.01 (**)

As shown in Fig. 4, host survival after infection was lower for *ea22* mutant phages with the overall effect being greater for the Stx phage Φ24.

**Fig. 5:**
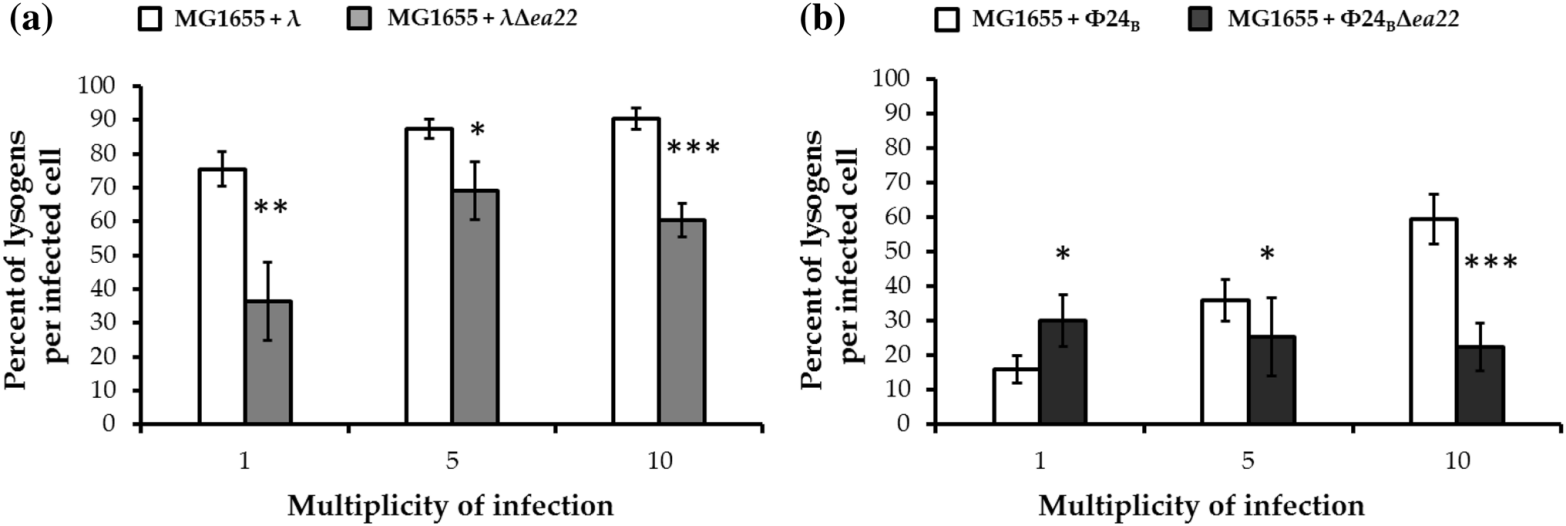
Percentage of lysogens per infected cell of *E. coli* MG1655 bacteria after infection with wild-type phages λ or Φ24_B_ (white columns) or their deletion mutants λΔ*ea22* (panel **a**; light gray columns) or Φ24Δ*ea22* (panel **b**; dark gray columns). Mean values ± SD from three biological experiments are shown. A *t* test was performed for results from each m.o.i. Statistically significant differences between wild-type phage and its deletion mutants are marked by asterisks, P < 0.05 (*) or P < 0.01 (**) or P < 0.001 (***)

Considering the formation and maintenance of lysogens, the overall efficiency of this process was reduced over a tenfold range of m.o.i. in λ *ea22* phage mutants (Table 4, Fig. 5). Interestingly, a different situation was observed in the case of Φ24_B_ phage. At m.o.i. 1 and 5, infection with Φ24_B_ mutant phage resulted in similar level of lysogens that was respectively higher (1) and lower (5) in comparison to wild-type phage. The opposite effects observed at both m.o.i. values were unexpected, though almost constant level of lysogens generated by the deletion mutant was also surprising. Importantly, this level of lysogens was persisted at the highest m.o.i. 10. It seems that the deletion of *ea22* gene alleviates the effect of increasing m.o.i. Again these effects were more pronounced for phage Φ24_B_.

**Table 4:** Percentage of lysogens among survived *E. coli* MG1655 bacteria infected with wild-type phages or their *ea22* deletion mutants

Strain/phage	Efficiency of lysogenization (% of lysogens among survivors)		
	m.o.i. = 1	m.o.i. = 5	m.o.i. = 10
MG1655+λ	48 ± 3	86 ± 3	90 ± 3
MG1655+λ∆*ea22*	23 ± 7	68 ± 8	60 ± 5
MG1655+Ф24_B_	10 ± 3	35 ± 6	59 ± 7
MG1655+Ф24_B_∆*ea22*	19 ± 5	25 ± 11	25 ± 7

## Discussion

The focus of this investigation was *ea22*, a relatively large gene in the *exo-xis* region that is common to both λ and Φ24_B_, a phage that we have used in this and prior studies as a representative of the Stx phages. From developmental assays of *ea22* mutant phages, we have observed that unlike other *exo-xis* genes such as *orf60a*, *orf63*, and *orf61,* expression of *ea22* favors the lysogenic over the lytic pathway and acts early in development. As *ea22* from both λ and Φ24_B_ is considered to resist lytic development, Stx phages may possess additional genes that work in conjunction with *ea22* to produce stronger effect than what we observed for λ bacteriophage. While two-hybrid studies of phage–host [33] and phage–phage [34] proteins failed to identify any interactions involving Ea22, they could have been impeded by low expression levels of one partner, weak interactions, the possibility that Ea22 acts in a multiprotein complex, or the possibility that Ea22 is a nucleic acid binding protein.

We suppose, *ea22* may act as key regulator of phage lysis vs. lysogeny decision in response to changing ratio of phage particles to bacterial cells. Although the presence of *ea22* favored lysogeny in most cases, the only deviation was observed in Stx phage at the lowest applied m.o.i. Based on it, we speculate that there is a putative additional regulation at m.o.i value = 1 that is considered as low and favorable for the lytic development of phages. It appears that additional factor(s) may interact with *ea22* under such conditions, abolish its action and even deepen the lytic response. Importantly, this additional regulation seems to be used only by selected phages. Taken together, *ea22* serves a role in decision to choose lytic or lysogenic pathway with the observed effects on phage development and host survival being greater for Stx phage.

Sequence analysis of *ea22* from λ and Stx phages offer some insight into the functional differences between λ and Stx phages. Among the Ea22 proteins presented, the carboxy terminus of the sequence (starting at 102 amino acid in λEa22) is dissimilar. However, all proteins terminate with a common K/RGE motif. We speculate that Stx^−^ and Stx^+^ phages have adopted a repertoire of carboxy-terminal domains that may all act upon the same host process but not necessarily the same host proteins. In addition to this, we observed an occurrence of a α-helical coiled-coil region in the N-terminal part of all the analyzed Ea22 protein sequences. Interestingly, a large number of the identified phage integrases (including λ Int) have extended coiled-coil domains which on the one hand, facilitate association and stabilization of the initial complex of recombination, and on the other hand, prevent prophage excision in the absence of the main excision protein [35].

The sequence divergence also extends to the expression patterns of *ea22* in λ and Stx phages during infection of *E. coli* host. With reference to λ phage, the level of mRNA for *ea22* is significantly lower when compared with the number of transcripts for *orf60a*, *orf63*, and *orf61,* whereas in Φ24_B_ and other Stx phages, we observe the opposite relationship. In fact, the level of *ea22* gene expression in Stx phages (especially in P32, P27, and P22) was significantly higher than the expression of other tested genes (even the early genes like*, N* and *cro*). Surprisingly, in opposite to *ea22*, the other genes (both early and late) were expressed at almost equal level. As we know from previous report, Stx phage genes present different levels of expression which increase during time-course infection experiment [29]. Due to this, we suspect that the efficient expression of early genes, e.g., *N* and *cro* have probably not started yet in the tested phages P32, P27, and P22. Importantly, at the same time, the level of expression of *ea22* was high, suggesting that despite belonging to the *p*L operon, the *ea22* gene might be expressed earlier and regulated independently of the studied phage genes. Above that, the expression of two other phage genes *ea8.5* and *int* have been analyzed by us previously in the frame of similar but not exactly the same infection experiment [29]. The obtained results indicate that the *ea8.5* presents a level of expression similar to that observed for *ea22* leading us to speculate that *ea22* has a similar mechanism of regulation. On the other hand, expression of *int* was decreased compared to most of the analyzed genes from *p*L operon [29]. This was possible due to the occurrence of double control of the *int* expression during phage infection. As shown previously, product of the *int* gene is responsible for an integrative recombination of phage and bacterial DNA during lysogenic pathway and works in conjunction with Xis to achieve excision of prophage DNA after induction [36]. The *int* gene is located near the *ea22* gene and is transcribed from both its own promoter, *p*I, and the leftward major *p*L promoter [37]. The *p*I promoter is positively regulated by cII protein which operates at the early stage of lysogenic infection. Shortly after making the decision to transition into a lytic cycle, when *p*I is inactive either due to the absence of cII [38], or by other *exo-xis* region proteins including Ea8.5 [21], *int* expression may still occur via *p*L [39]. Looking for convergence in the regulation of the *int* and *ea22* genes expression, we came across the data from a comprehensive ribosomal profiling study of phage genes during early lytic infection. These data allowed us to identify *int* as a gene with a similar to *ea22* profile and level of expression in the first twenty minutes of temperature-dependent prophage induction [39]—Fig. 1. In addition, upstream of the λ and Φ24_B_*ea22* genes, there was a predicted but never investigated promoter named *p*1 [29]. Interestingly, the *p*1 promoter, like the well-established *p*I promoter, also contains binding sites for host RNA polymerase sigma factor 70 (RpoD17) and two host arginine-sensitive regulators ArgR and ArgR2, as found by BPROM software [29]. Undoubtedly, at this stage of knowledge, we cannot exclude that similar to that observed in *int,* the double control of *ea22* expression also exists*.* However, further research are needed to confirm the activity of *p*1 promoter and to explain the mechanism of this regulation.

The last aspect of this discussion focused on the physiological significance of *ea22* expression. We showed that in contrast to other *exo-xis* genes (*orf60a*, *orf63* and *orf61)*, expression of *ea22* favors the lysogenic over the lytic pathway. Wild-type λ and Φ24_B_ phages revealed higher efficiency of lysogenization of bacterial cells and lower efficiency of progeny phage production during the lytic cycle, when compared with Δ*ea22* mutants. Interestingly, differences between wild-type and mutant phages were more pronounced in Φ24_B_ than in λ. In the context of *ea22* gene expression, its disparate function relative to other *exo-xis* region genes, and some similarities in its mechanism of regulation to phage integrase, there is a possibility that Ea22 navigates the lysogenic-lytic decision by working in concert with the phage integrase and is dependent on the physiological state of the bacterial cell. It is worth noting that the function of *ea22* that we have proposed is different from earlier assumptions. In one of previous reports, authors concluded that Ea22 from λ is similar to Ehly 2 protein that is associated with an enterohemolysin 2 activity and encoded by phage C3208 in *E. coli* O26:H11 [40, 41]. This finding was based on the sequence similarity that was estimated at level of 35% [40]. According to the work of Rost and collaborators, such a level of identity is considered as result of twilight zone that is difficult to interpret [42]. In addition, the location of the *ea22* gene in the early region of the phage genome does not indicate on its enterohemolysin activity. Importantly, there were no other premises that indicated on similarity of Ea22 to Ehly2.

## Conclusion

We have presented a series of investigation that appear to show that, Ea22 has a different function than previously assumed. Unlike other analyzed genes of the *exo-xis* region [30, 31], *ea22* is a functional gene whose product acts to favor the lysogenic state over lytic infection, possibly by the kind of host protein partnerships it makes. The regulation of *ea22* seems to be distinct from other *exo-xis* region genes, and thus more similar to the *int* gene. Using *ea22* deletion mutants in λ and several Stx phages including Φ24_B_, we have observed developmental differences that may arise due to a divergent carboxy-terminal sequence in each protein. Our work to date suggests that *ea22* represents an important new gene to study aspects of lysogenic-lytic decision. Based on this, Ea22 could serve as a new target for potential therapy against STEC infections. Its prolysogenic effect could be used in the future to inhibit the lytic development of Stx phages and thereby to limit the production of dangerous to human health Shiga toxins.
